# Weight-Based PA-GPSR Protocol Improvement Method in VANET

**DOI:** 10.3390/s23135991

**Published:** 2023-06-28

**Authors:** Wenzhu Zhang, Leilei Jiang, Xi Song, Zhengyuan Shao

**Affiliations:** School of Information and Control Engineering, Xi’an University of Architecture and Technology, Xi’an 710055, China; jiangleilei@xauat.edu.cn (L.J.); xisong@xauat.edu.cn (X.S.); shaozhengyuan@xauat.edu.cn (Z.S.)

**Keywords:** VANET, routing protocol, path aware, greedy strategy, perimeter strategy

## Abstract

Vehicle Ad-hoc network (VANET) can provide technical support and solutions for the construction of intelligent and efficient transportation systems, and the routing protocol directly affects the efficiency of VANET. The rapid movement of nodes and uneven density distribution affect the routing stability and data transmission efficiency in VANET. To improve the local optimality and routing loops of the path-aware greedy perimeter stateless routing protocol (PA-GPSR) in urban sparse networks, a weight-based path-aware greedy perimeter stateless routing protocol (W-PAGPSR) is proposed. The protocol is divided into two stages. Firstly, in the routing establishment stage, the node distance, reliable node density, cumulative communication duration, and node movement direction are integrated to indicate the communication reliability of the node, and the next hop node is selected using the weight greedy forwarding strategy to achieve reliable transmission of data packets. Secondly, in the routing maintenance stage, based on the data packet delivery angle and reliable node density, the next hop node is selected for forwarding using the weight perimeter forwarding strategy to achieve routing repair. The simulation results show that compared to the greedy peripheral stateless routing protocol (GPSR), for the maximum distance–minimum angle greedy peripheral stateless routing (MM-GPSR) and PA-GPSR protocols, the packet loss rate of the protocol is reduced by an average of 24.47%, 25.02%, and 14.12%, respectively; the average end-to-end delay is reduced by an average of 48.34%, 79.96%, and 21.45%, respectively; and the network throughput is increased by an average of 47.68%, 58.39%, and 20.33%, respectively. This protocol improves network throughput while reducing the average end-to-end delay and packet loss rate.

## 1. Introduction

As the number of automobiles in the world continues to increase, countries around are also paying ever-more attention to traffic congestion and accidents. The intelligent transport system (ITS) proposed to effectively improve traffic efficiency plays an extremely important role in the mobility of today’s digital world [1]. VANET is a special kind of mobile ad hoc network (MANET), which is widely used in the transportation field and has become an important part of ITS [2,3]. With the rapid growth in network technology, computing power, and storage resources, VANET has become a network with continuous self-configuration and no infrastructure characteristics [4]. VANET can provide onboard services, such as information about traffic, road accidents, weather conditions, and general traffic warnings [5,6]. Vehicles that have encountered a road accident can broadcast news about this incident. Other vehicles can change their driving routes in advance to avoid possible traffic jams or other accidents, thereby improving the driving experience and safety. Each vehicle in VANET is equipped with an onboard unit (OBU), which is a built-in onboard network device that can share real-time traffic-related information with surrounding vehicles and distributed roadside units (RSU) [7]. The communication modes in VANET can be divided into vehicle-to-vehicle (V2V) and vehicle-to-infrastructure communication (V2I) [8,9]. Due to the cost of installing RSU, it is not feasible to use a large number of RSUs to provide the entire network connection. Reliable routing schemes can be used to improve the efficiency of data dissemination in a V2V environment.

The routing protocol is an important part of VANET, and its performance directly determines the efficiency of VANET. However, due to the continuous changes in the network topology and the mobility of vehicle nodes, the success rate of data packet transmission cannot be guaranteed, resulting in the routing protocol previously applicable to MANET being no longer applicable to VANET [10,11]. As there is no need to maintain any routing table or share connection status with neighbors, routing is only established when forwarding data packets. Geographic location-based routing protocols are considered more suitable for VANET [12,13]. Since the global positioning system (GPS) is widely used in vehicles, the location of the vehicle can be easily obtained through periodic beacon.

In summary, the existing location-based research improves the selection of optimal forwarding nodes in different ways. However, due to the high topology changes in VANET, these schemes will also encounter performance degradation problems. Given the complex characteristics and application requirements of sparse network scenarios in urban environments, this paper proposes a weight-based path-aware greedy peripheral stateless routing protocol (W-PAGPSR). In the routing establishment stage, based on the distance, direction, reliable node density, and cumulative communication duration, the greedy forwarding strategy is used to select the next hop node to reduce the problem of poor performance in sparse networks caused by frequent link interruptions and data retransmissions. In the routing maintenance stage, according to the left and right-hand rules, the data packet delivery angle and reliable node density are considered comprehensively, and the next hop node is selected using the perimeter forwarding strategy to improve the high delay caused by the routing loop. Finally, the data packet is delivered to the destination through multi-hop transmission, which achieves the effect of reducing end-to-end delay, increasing throughput, and improving the packet delivery rate.

The main contributions of this paper are as follows:

(1) We improve the location-based routing protocol in VANET.
(2) Two node selection strategies based on weight are proposed, and the next hop node is selected according to the following parameters: reliable node density, node location, node movement direction, node cumulative communication duration, and data packet delivery angle.
(3) We study the impact of the number of different nodes on performance through the proposed strategy.

The rest of this paper is arranged as follows: the second section introduces related works, the third section introduces the formation of W-PAGPSR, the fourth section describes the improved forwarding mechanism in detail, the fifth section introduces the simulation results and analysis, and the sixth section summarizes the work of this paper and the scope of future work.

## 2. Related Work

At present, many research have been proposed to improve the selection of forwarding nodes for geographic location routing protocols. In the geographic perimeter stateless routing protocol (GPSR) proposed by Karp et al. [14], the source node forwards the data packet to the neighbor node closest to the destination. When the source node does not find any neighbor node closer to the destination node, the perimeter forwarding strategy based on the right-hand rule is used for transmission. Due to its stateless routing, GPSR may encounter a situation where the route is disconnected and the message delivery fails. The GPSR+ predict protocol (GPSRJ+) proposed by Houssaini et al. [15] uses the estimated future location of the neighbor node to select the next hop node and improves the greedy forwarding and perimeter forwarding strategies of GPSR. Both protocols regard the distance to the destination as the only indicator for forwarding data packets, which are prone to routing voids. Yang et al. [16] proposed the MM-GPSR, which uses pre-defined λ parameters in greedy forwarding to control the communication area in which to find the next hop node and uses the minimum angle value in perimeter forwarding to select the next hop. However, its pre-defined parameters reduce the performance of the scheme. Silva et al. [16,17] proposed PA-GPSR, adding additional information to the location table, as well as using the rejection table and the most recent forwarding table to mark the nodes where routing voids occur to reduce the number of data retransmissions and achieve the purpose of reducing network overhead. However, PA-GPSR does not use basic routing indicators, such as link connectivity and relative speed, and the data packets copied in perimeter forwarding increase network overhead. The predictive geographic routing protocol (PGRP) proposed by Karim et al. [18] selects the next hop according to the direction, predictive position, and angle of neighbors. The Kalman filter-predictive geographic routing protocol (K-PGRP) proposed by Punia et al. [19] wields the Kalman filter as a prediction module in the PGRP routing protocol. The mobility prediction-based routing protocol (MPBRP) proposed by Ye et al. [20] improved the greedy forwarding strategy and perimeter forwarding strategy based on predictive position. These studies did not consider the impact of neighbor node density and link reliability. Gu et al. [21] proposed the weighted greedy perimeter coordinator routing protocol (W-GPCR) based on weight selection, which calculates the next hop node in greedy forwarding and perimeter forwarding by considering the multi-parameter formula designed based on distance, direction, and neighbor node density. The research considers the influence of all neighbor nodes found in the communication range. The Hybrid Relay Node Selection Scheme for Message (HRNS) protocol proposed by Osama et al. [22] uses message reachability, communication delay, and bandwidth utilization to select the next hop node, though while increasing the packet arrival rate, it also increases the end-to-end delay.

The Geo-LU proposed by Ohoud et al. [23] selects the next hop node based on the two-hop neighbor information, the minimum remaining bandwidth, and its packet loss rate. The trust-based geographic routing protocol for VANETs (TGRV) proposed by Saeed et al. [24] uses trust to evaluate the packet forwarding rate while considering the distance, speed, and direction of neighbors, and it selects the next hop node based on the information and trust of the two-hop neighbors. The smart forwarding method-based data dissemination protocol (SFTD) proposed by Mahesh et al. [25] evaluates the link quality based on the link duration and the received signal strength indication and selects the next hop node. Bidisha et al. [26] proposed reliable and efficient RSU-enabled relay vehicle selection protocol (ReUse) to select the next hop node based on various parameters, such as mobility, link quality, buffer size, and the number of neighbors. The Obstacle Prediction-Based Routing Protocol (OPBRP) proposed by M. Khalid et al. [27] uses mobility prediction and mobility to improve the prediction-based routing protocol to select the next hop node. The cross-layer enhancement of geographic routing protocol (Geo-CAP) proposed by Ohoud et al. [28] considers the packet loss rate, physical interference, and media access control layer overhead to establish a link model and select the next hop node. The multi-criteria-based broadcast suppression scheme protocol (MCBS) proposed by Yonas et al. [29] uses multiple node attributes, including relative speed, traffic density, link stability, movement angle, and distance to the sender, to estimate the quality of the node and select the next hop node. A position-based reliable emergency message routing (REMR) protocol proposed by Ghulam et al. [30] uses vehicle motion information (for example, position, speed change, and movement angle) to minimize possible link interruptions and select the next hop node. Zhou et al. [31] proposed a multi-intersection selection based on the probability routing protocol (MISP) of the road section connection. This protocol considers the distribution of vehicles affected by traffic lights and completes the data transmission by selecting the optimal path and the relay node in the road section. The trunk line based geographic routing protocol (TLBGR) proposed by Chen et al. [32] uses the traffic flow of the trunk line and the surrounding road network to provide real-time data, and it completes the data transmission through the selection of the next intersection and the next hop of the selected path between the current and the next intersection. The improved road segment-based geographical routing protocol (ISR) proposed by Qureshi et al. [33] divides the forwarding area into a number of road segments and selects a head node on each segment by focusing on the location, direction, and link quality-centric score for every vehicle on each road segment. These studies mainly consider node motion and channel state for link performance modeling to improve location-based routing protocols. These protocols are prone to routing voids in sparse networks, resulting in high transmission delay. In addition, the computational complexity of some studies is high and is not suitable for sparse network scenarios.

The relay node selection mechanism of routing protocols can be divided into sender- or receiver-oriented protocols [34,35]. Table 1 presents a summary of reviewed location-based routing protocols based on their taxonomy, with most of them considered to be sender-oriented protocols.

## 3. Formation of Weighted Path Aware Greedy Perimeter Stateless Routing Protocol

### 3.1. Influence of Node Movement Direction on Communication Reliability

**Figure 1:**
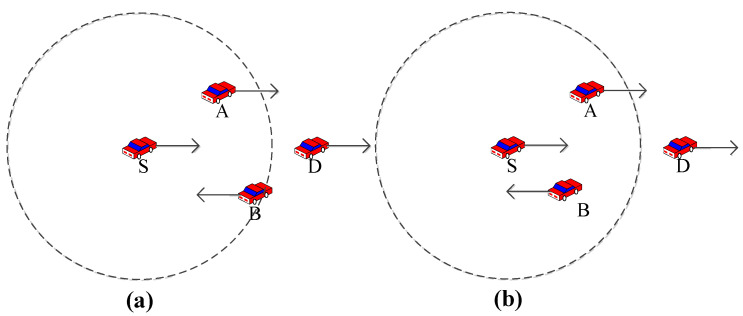
The influence of node movement direction on communication reliability. A schematic diagram of packet delivery: (**a**) before packet delivery at T moment; (**b**) after packet delivery at T + 1 moment. In the figure, node S is the source node, nodes A and B are relay nodes, node D is the destination node, the dotted circles represent the communication range, and the solid line and arrows represent the direction of node movement.

As shown in Figure 1a at T moment, the source node S sends a data packet to the destination node D. The direction of movement of node B is opposite to those of nodes S, A, and D. In the traditional greedy forwarding strategy, node B is closer; thus, node S selects node B for data transmission. However, as shown in Figure 1b at T + 1 moment, the node location changes, which will cause additional end-to-end delays, because node B will again select node A as the next hop node after receiving the packet. Therefore, when choosing the next hop node, not only does the distance need to be considered, but the direction of movement of the node also needs to be considered. Node S selects node A for data transmission, which will reduce the packet loss rate and average end-to-end delay.

### 3.2. Influence of Reliable Node Density on Communication Reliability

**Figure 2:**
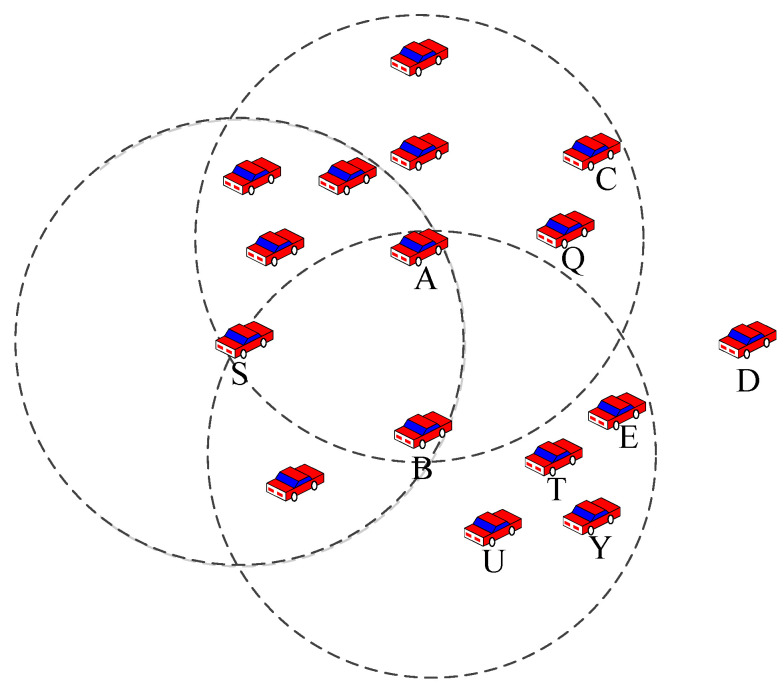
The influence of reliable neighbor node density on communication reliability. The reliable neighbor nodes of node A are nodes C and Q, and the reliable neighbor nodes of node B are nodes U, T, E, and Y.

As shown in Figure 2, when node S transmits data to destination node D, its neighbor nodes A and B are the same distance from destination node D. If the judgment is based on the density of all neighbor nodes within the communication range, at this time, the densities of neighbor nodes of node A are greater than those of node B; thus, the data packet should be transmitted to node A. However, during data packet transmission, only nodes in the reliable communication area contribute to the communication reliability, such as node Q. The reliable neighbor nodes of node A are nodes C and Q, and the reliable neighbor nodes of node B are nodes U, T, E, and Y; thus, the reliable node density of node B is higher. Node S forwards data to node B, and the probability of establishing a reliable routing link in the future is greater, thereby reducing the probability of losing data packets.

### 3.3. Influence of Cumulative Communication Duration on Communication Reliability

The cumulative communication duration defines the stability of the communication link between the neighbor node and the node currently carrying the data packet [15,16]. In Figure 2, nodes Q and C are in the reliable communication area of node A, and, thus, the cumulative communication duration between the two neighbor nodes and node S is calculated as follows:

(1) $$T_{i}=T_{i-1}+t_{i}-t_{i-1}$$

In Formula (1), $T_{i}$ is the current cumulative communication duration, $T_{i-1}$ is the last cumulative communication duration, $t_{i}$ is the current time of receiving the HELLO packet, and $t_{i-1}$ is the time of receiving the last HELLO packet. If $T_{1}=0$, $t_{1}$ is the moment of receiving the first HELLO packet. By comparison with nodes Q and C, the node that has the largest $T_{i}$ will be selected as the next hop node of S. When forwarding data packets according to this method, the greater the cumulative communication duration, the higher the possibility of establishing reliable communication between the two nodes, as well as the smaller the probability of data loss.

By introducing reference factors, such as the direction of movement of the next hop node and the density of reliable nodes, this paper optimizes the traditional greedy forwarding strategy. Compared to simply judging the distance between the next hop node and the destination node, this optimization strategy can reduce the limitations of the strategy and provide a better solution.

**Figure 3:**
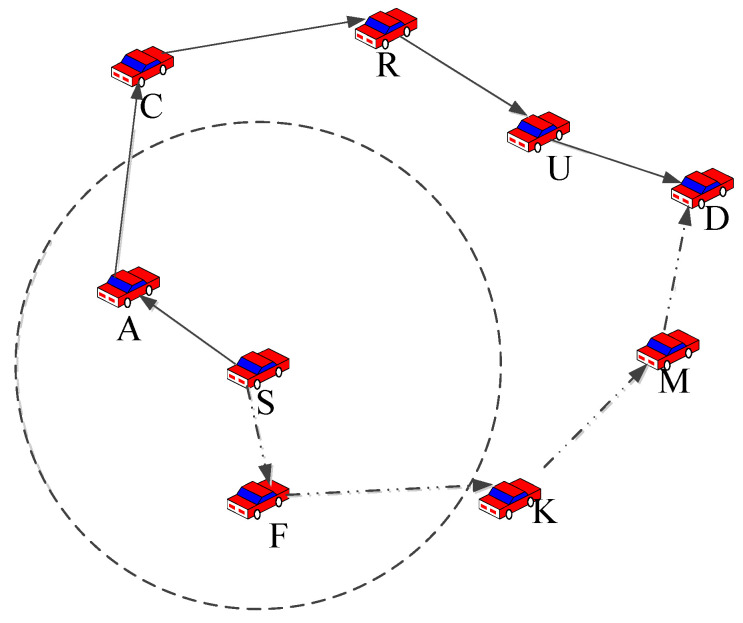
A schematic diagram of perimeter rule forwarding packets. The construction path devised via a traditional strategy using arrows with solid lines is S-A-C-R-U-D, and the construction path devised via an improved strategy using arrows with dashed lines is S-A-C-R-U-D.

When the node forwarding the data packet is closer to the destination node than all of its neighbor nodes in location-based routing protocols, and if the destination node does not exist within its transmission range, the greedy forwarding strategy will fail, and at this point, the routing protocol is said to be trapped in a routing void. To overcome this problem, GPSR and other traditional greedy routing protocols will be converted to traditional perimeter forwarding strategies for maintenance. The traditional perimeter forwarding strategy uses the right-hand rule and transmits data packets around the periphery of the area until it reaches an adjacent node located closer to the destination node. For example, Figure 3 shows that within the communication range of node S, no node is closer to the destination node D than S. Therefore, node S adopts the right-hand rule, and the construction path is S-A-C-R-U-D. However, if the angle of the packet delivery direction and the angle of the node S pointing to the destination node D and the reliable node density are considered, node S will select node F as the next hop, and the construction path will be S-F-K-M-D, which reduces path redundancy. By comparing the two paths, it is found that the path in the traditional perimeter forwarding strategy is longer, which increases the number of hops and produces a higher delay.

This paper optimizes networks on the basis of the traditional perimeter forwarding strategy, taking the delivery direction of data packets as the main reference quantity, to be supplemented by the density of reliable nodes, meaning that the direction of data packet delivery converges on the direction of the destination node, thereby reducing the path redundancy and routing loop probability caused by the traditional perimeter forwarding strategy.

## 4. Proposed Protocol

This section specifically introduces the proposed routing protocol. W-PAGPSR uses nodes to create paths on demand, uses real-time GPS information to monitor them, and maintains a list of all neighbor nodes within the range of the node. Under the existing system, the IP address of the vehicle is the unique identification marker of each node; thus, the list maintained by the node consists of IDs. At this time, when a node is designated as the source node carrying the data packet, the greedy and perimeter weights of the reliable node are calculated in the neighbor table, which depends on the five parameters between the sender and all reliable nodes, and the data packet is then transmitted.

### 4.1. System Model and Assumptions

**Figure 4:**
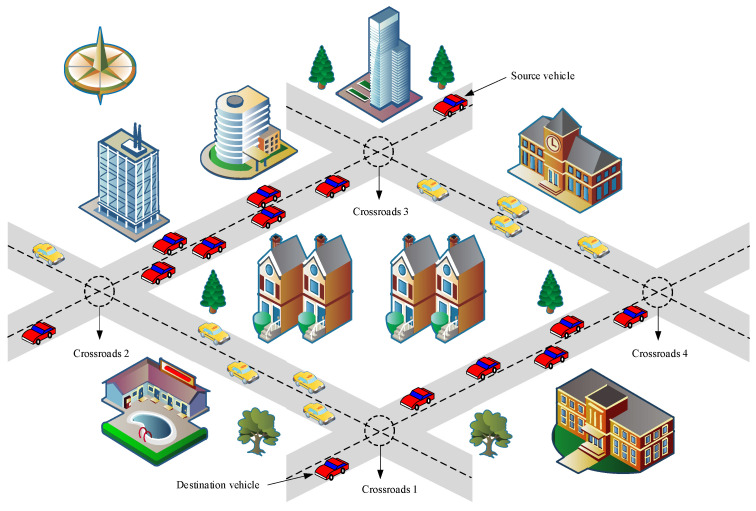
A system scenario diagram, in which the dashed circles represent the intersections, and the dotted lines represent the lane boundaries.

(1) Location and radius: each vehicle was equipped with a Global Positioning System (GPS) and OBU, which have fixed accessible communication ranged [36]. OBU enabled the vehicle to send a beacon within its communication range to perform inter-vehicle communication. In W-GPSR, similar to most geographic routing protocols, it was assumed that there is a centralized location service. Each forwarding node could obtain destination location information by querying the central management organization to meet routing requirements.
(2) Communication and sensors: each vehicle was equipped with a wireless network interface that complies with 802.11p or dedicated short-range communication standards for inter-vehicle communication [34]. In addition, each vehicle was also equipped with an onboard diagnostic interface, which was designed to obtain data from multiple mechanical and electronic sensors in the vehicle. There was no infrastructure around the road, and nodes can only exchange data through V2V.
(3) Neighbors and paths: the initial location of the vehicle was determined based on a random selection of uniform distribution. When vehicles were within communication range of each other, an edge connection was established between their neighbors. Communication could be established based on direct contact with each other or through neighbors. Due to the dynamic nature of VANET, communication had to be established iteratively using dynamic routing protocols.
(4) Nodes share information: these data included information about modes’ state vectors, such as physical location, destination, and direction.
(5) Model area: the model area was constrained by the plane frame. The network model consisted of roads and intersections that simulated a typical urban environment. The system scenario diagram is shown in Figure 4.
(6) The location information provided by the positioning system was accurate, i.e., the location error was not considered.

### 4.2. System Architecture and Data Structure

**Figure 5:**
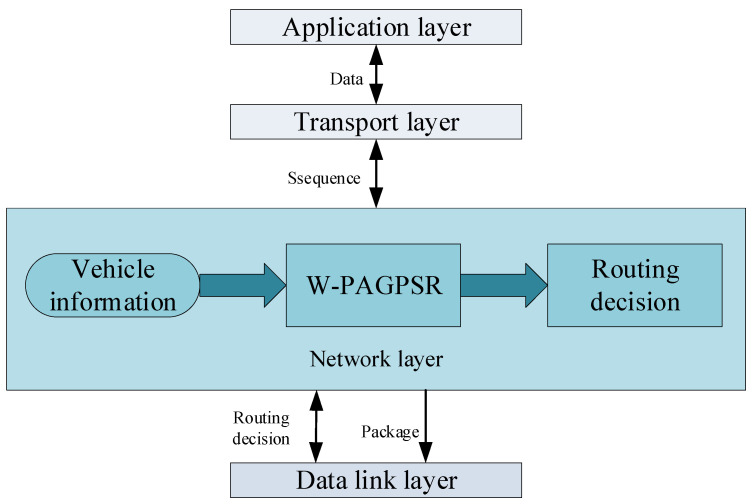
A system architecture diagram.

The system architecture of W-PAGPSR is shown in Figure 5:

HELLO messages were generated periodically at pre-defined times to avoid synchronization and potential conflicts. Table 2 shows the format of the HELLO message used in W-PAGPSR, and Table 3 shows the format of the data packet used in W-PAGPSR.

### 4.3. Vehicle Follow-Up Model

We used the improved safe distance Krauss model [37] of the Simulation of Urban Mobility (SUMO) [38] as the vehicle follow-up model. The speed of the vehicle is limited based on the safe speed $r_{\mathrm{safe}}$ as follows:

(2) $$r_{safe}=r_{previous}+\frac{d-r_{current}\tau }{(r_{previous}+r_{current})/2a+\tau }$$

In Formula (2), $r_{\mathrm{previous}}$ is the speed of the front vehicle; d is the distance between two vehicles; $r_{\mathrm{current}}$ is the speed of the current vehicle; a is maximum vehicle deceleration; and τ is the driver’s reaction time.

### 4.4. Packet Queuing Model

During simulation, the first-in, first-out (FIFO) strategy was used to manage the transmission and reception of data packets. When the queue length exceeded the pre-set value, the queue performed packet loss operations on subsequent packets through the tail loss strategy. The data packet is discarded as follows:

(3) $$t_{now}\geq t_{in}+t_{expire}$$

In Formula (3), $t_{\mathrm{now}}$ is the current time; $t_{\mathrm{in}}$ is the time when the packet enters the queue; and $t_{\mathrm{expire}}$ is the allowed survival time for data packets. When the conditions were met, expired packets in the queue were deleted.

The W-PAGPSR proposed in this paper was a location-based V2V routing protocol, the purpose of which was to improve the greedy and perimeter forwarding strategies of PA-GPSR through link stability.

### 4.5. Greedy Forwarding Route Establishment Strategy Based on Weight

The weight of the strategy proposed in this section changed according to the changes in the neighbor table in VANET; thus, the proposed routing protocol was adaptive. This section comprehensively considered the distances between nodes and the direction of the movement of nodes, combined with the reliable node density and the cumulative communication duration of nodes, with the weight calculation method proposed as follows:

(4) $$G_{n}=w_{1}(\frac{D_{sd}-D_{nd}}{D_{sd}})+w_{2}\cos\theta +w_{3}\frac{RN_{n}}{S}+w_{4}T_{i}$$

In Formula (4), $G_{n}$ defines the weight value of the node n. $D_{\mathrm{nd}}$ is the Euclidean distance between the node n and the destination node d, while $D_{\mathrm{sd}}$ is the Euclidean distance between the source node s and the destination node d. θ is the angle between the speed vector $V_{n}$ of the node n and the direction vector $L_{\mathrm{nd}}$ of the node n pointing to the destination node. ${\mathrm{RN}}_{n}$ is the number of reliable neighbor nodes of the node n, while S is the area of the communication range of node n. $w_{i}$ is the weight coefficient, $w_{1}{+w}_{2}{+w}_{3}{+w}_{4}=1$ and $w_{1}{,w}_{2}{,w}_{3}{,w}_{4}\in \left[0,1\right]$.

The Euclidean distance $D_{\mathrm{sd}}$ between the two nodes is defined as follows:

(5) $$D_{sd}=\sqrt{{\left(x_{s}-x_{d}\right)}^{2}+{\left(y_{s}-y_{d}\right)}^{2}}$$

The angle θ between the two nodes is defined as follows:

(6) $$\theta =\cos^{-1}(\frac{\overset{}{V_{n}}\times \overset{}{L_{nd}}}{∥\overset{}{V_{n}}∥\times ∥\;\overset{}{L_{nd}}∥})$$

In Formula (6), $V_{n}$ is the speed vector of node n, while $\overset{}{L_{\mathrm{nd}}}$ is the direction vector of node n pointing to the destination node d. The larger the cosθ value, the closer the direction of movement of the node is to the direction of movement of the destination node.

The cumulative communication duration of node n for node s is defined in Formula (1), i.e., the larger the $T_{i}$ value, the closer the communication between the nodes n and s.

Formula (7) can be used to determine whether a neighbor node is a reliable node:

(7) $$D_{sd}-D_{nd}\geq 0$$

We calculated the reliable node density via formula (8):

(8) $$\frac{RN_{n}}{S}=\frac{RN_{n}}{\pi r^{2}}$$

### 4.6. Perimeter Forwarding Route Maintenance Strategy Based on Weight

In this section, a weight calculation method based on the data packet delivery angle and assisted by reliable node density was proposed to realize the improvement of the PA-GPSR protocol’s perimeter forwarding strategy. The perimeter forwarding strategy of the PA-GPSR protocol was to use the left-hand and right-hand rules for forwarding while copying data packet, as well as to use the recently sent table to reduce the overhead of the routing protocol, i.e., those that had already forwarded left-handed data packets could no longer forward right-handed data packets. The weight-based perimeter forwarding strategy reduces redundant paths and further reduces end-to-end delay. The specific weight calculation method is defined as follows:

(9) $$P_{n}=\beta_{1}\cos\theta_{n}+\beta_{2}\frac{RN_{n}}{S}$$

In Formula (9), $\theta_{n}$ is the data packet delivery angle, while $\beta_{i}$ is the weight coefficient, i.e., $\beta_{1}{+\beta }_{2}=1$ and $\beta_{1}{,\beta }_{2}\in \left[0,1\right]$. The calculation formula of $\theta_{n}$ is defined via Formulas (10) and (11):

(10) $$\cos\theta_{n}=\frac{\left|y_{i}-y_{s}\right|}{\sqrt{{\left(x_{i}-x_{s}\right)}^{2}+{\left(y_{i}-y_{s}\right)}^{2}}}$$

(11) $$\theta_{n}=\left\{\begin{array}{l} \theta_{right}=arccos(cos\theta_{i}),node\;i\;is\;in\;the\;first\;quadrant\\ \theta_{left}=arccos(cos\theta_{i}),node\;i\;is\;in\;the\;second\;quadrant\\ \theta_{left}=\pi -arccos(cos\theta_{{}_{i}}),node\;i\;is\;in\;the\;third\;quadrant\\ \theta_{right}=\pi -arccos(cos\theta_{i}),node\;i\;is\;in\;the\;forth\;quadrant \end{array}\right.$$

**Figure 6:**
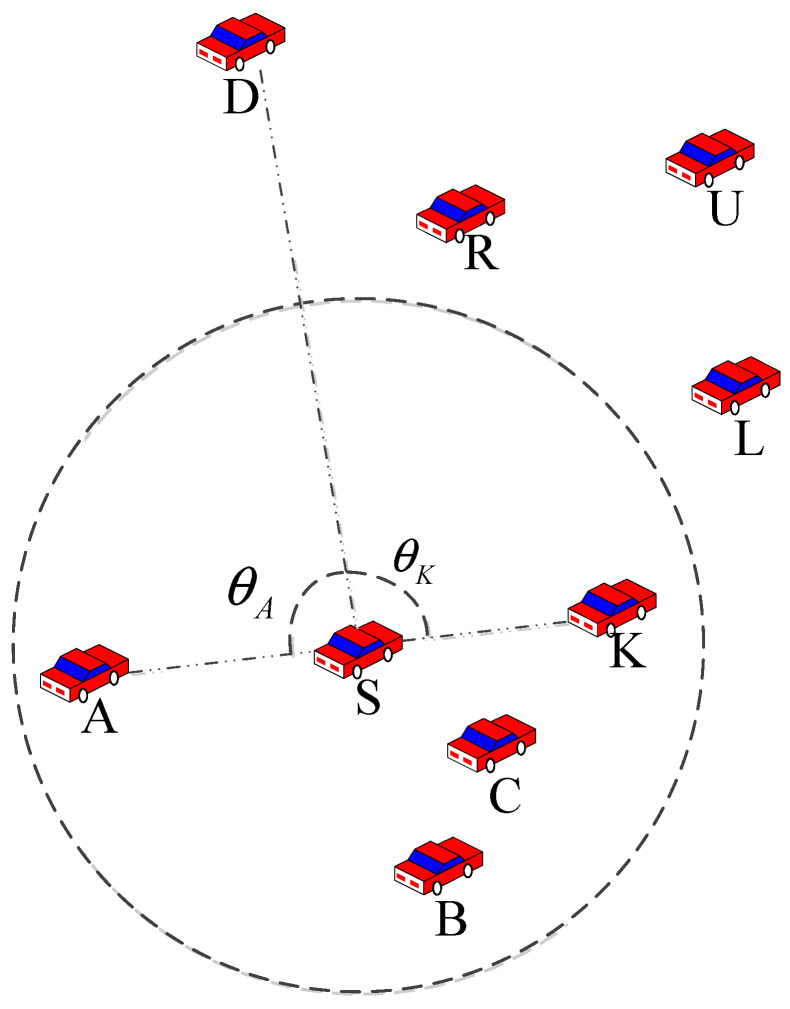
A schematic diagram of improved perimeter forwarding based on packet delivery angle and reliable node density. In the figure, $\theta_{A}$ and $\theta_{K}$ are packet delivery angles, and nodes R, U, and L are reliable neighbors of node K.

As shown in Figure 6, we first calculated the packet delivery angle and reliable node density of node S for nodes A, K, D, and B, and then select node K as the next hop node based on weight. The packet delivery angle of the neighbor nodes in the left and right halves of the plane was calculated, and the weight was then determined based on the density of the reliable nodes; the next relay node for perimeter forwarding was determined based on the weight.

The weight formula proposed in this section was calculated using multiple indicators; thus, a reliable weight distribution method is needed. The CRITIC method comprehensively measures the objective weight of indicators and uses the objective attributes of the data to evaluate them. It is an effective objective empowerment method [39].

Firstly, we obtained the relevant data to form the original data matrix X, where X(m,n) represented the n·th item of the m·th sample. The values of the evaluation indicators are defined as follows:

(12) $$X=\left(\begin{array}{ccc} X_{1,1} & \ldots & X_{1,n}\\ \vdots & \ddots & \vdots \\ a_{m,1} & \ldots & a_{m,n} \end{array}\right)$$

To eliminate the effects of inconsistent dimensions, dimensionless processing was carried out. The forward indicator was selected, and the weight coefficient selected is shown in Formula (13):

(13) $$x_{ij}^{\prime }=\frac{max(x_{j})-x_{ij}}{max(x_{j})-min(x_{j})}$$

Before calculating the amount of information $C_{j}$, we first calculated the volatility $S_{j}$, as shown in Formula (14), where $X_{j}$ is the mean of each indicator, and $S_{j}$ is the standard deviation of the j·th indicator.

(14) $$x_{ij}^{\prime }=\frac{max(x_{j})-x_{ij}}{max(x_{j})-min(x_{j})}$$

The correlation matrix R of the indicator was used to calculate the conflict, as shown in Formula (15):

(15) $$R=\frac{\sum {}_{j,k=1}^{n}(x_{ij}-{\overset{-}{x}}_{j})(x_{ik}-{\overset{-}{x}}_{k})}{\sqrt{\sum {}_{j=1}^{n}{\left(x_{ij}-{\overset{-}{x}}_{j}\right)}^{2}{\sum {}_{k=1}^{n}(x_{ik}-{\overset{-}{x}}_{k})}^{2}}}$$

We then calculated the conflict $A_{j}$, as shown in Formula (16), where $r_{\mathrm{ij}}$ represented the correlation coefficient of the i·th and the j·th indicators. The stronger the correlation with other indicators, the lesser the conflict and the greater the weight.

(16) $$A_{j}=\sum {}_{i=1}^{n}(1-r_{ij})$$

The greater the amount of calculated information $C_{j}$, the greater the weight of the j·th evaluation index in the entire evaluation index system, as shown in Formula (17):

(17) $$C_{j}=S_{j}\times A_{j}$$

Finally, we calculated the weight coefficient $W_{j}$, as shown in Formula (18):

(18) $$W_{j}=\frac{C_{j}}{\sum {}_{j=1}^{n}C_{j}}$$

### 4.7. The Overall Process of the Protocol

**Figure 7:**
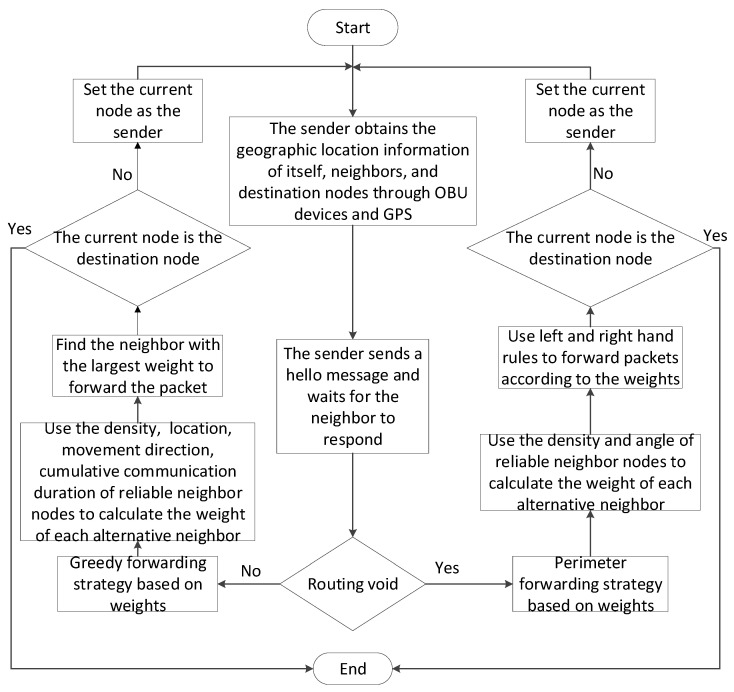
A flow chart of the proposed protocol.

The process of the W-PAGPSR is shown in Figure 7. The sender or source node was first sent to obtain the location information of the neighbor node through the OBU device and GPS, before sending a HELLO message and waiting for the node to respond. The next step was to calculate the Euclidean distance from each neighbor node to the destination node in the communication range to determine the reliable neighbor node. We then checked for routing voids. If they were present, we used the weight-based perimeter forwarding strategy. If they were not present, we used the following steps: we calculated the angle between each neighbor and the destination node within the reliable communication range, the reliable node density of the neighbor, the cumulative communication duration between each neighbor, and the sender or source node, and, finally, calculated the weight of each candidate neighbor and selected the best relay node to deliver the message. In the perimeter forwarding strategy where the greedy forwarding strategy failed, the current node first calculated and compared the perimeter weight of the neighbor node, and then selected the neighbor node with the largest weight in the left and right half planes as the next hop to forward the packet. We executed Algorithm 1 when the data packet was transmitted.
**Algorithm 1:** W-PAGPSR.**Input:** Source node $N_{C}$; Destination node $N_{D}$; **Output:** Best relay node; 1. **While** $N_{C}$ is the receive packet, p is 2.  **If** $N_{C}{==N}_{D}$, **then** 3.    The best relay node is $N_{C}$; 4.  **If** p is HELLO packet, **then** 5.    Use the content in p to update or create the content in NT, such as the number of neighbors, location, co-ordinates, speed, etc. 6.  **End if** 7.  **If** p is the data packet, **then** 8.    **If** using greedy forwarding mode, **then** 9.      Calculate $D_{\mathrm{cd}}$ and $D_{\mathrm{neighbor}\to d}$ according to formula (5) 10.        **For** (All neighbors $N_{\mathrm{neighbor}}$ in NT and $D_{\mathrm{neighbor}\to d}\leq D_{\mathrm{cd}}$) **do** 11.          Calculate angle $\theta_{\mathrm{neighbor}}$ according to Formula (6) 12.          Calculate $T_{\mathrm{neighbor}}$ according to Formula (1) 13.          Calculate ${\mathrm{RN}}_{n}/S$ according to Formula (8) 14.          Calculate $G_{\mathrm{neighbor}}$ according to Formula (4) 15.          **If** $G_{\mathrm{neighbor}}{>G}_{n}$**then** 16.            $G_{\max}\leftarrow G_{\mathrm{neighbor}}$ 17.            $N_{\mathrm{next}}\leftarrow N_{\mathrm{neighbor}}$ 18.          **End if**
19.        **End for** 20.      Update p and then forward p to $N_{\mathrm{next}}$ 21.    **Else** 22.      **For** (All neighbors $N_{\mathrm{neighbor}}$ in NT), perform the following actions 23.        Calculate $\theta_{\mathrm{left}}$ and $\theta_{\mathrm{right}}$ according to Formulas (10) and (11) 24.        Calculate ${\mathrm{RN}}_{n}/S$ according to Formula (8) 25.        Calculate $P_{\mathrm{neighbor}-\mathrm{left}}$ and $P_{\mathrm{neighbor}-\mathrm{right}}$ according to Formula (9) 26.        **If** $N_{\mathrm{neighbor}}$ is in the left half plane and $P_{\mathrm{neighbor}-\mathrm{left}}{>P}_{\mathrm{left}}$, **then** 27.          $P_{\mathrm{left}}\leftarrow P_{\mathrm{neighbor}-\mathrm{left}}$ 28.          $N_{\mathrm{next}-\mathrm{left}}\leftarrow N_{\mathrm{neighbor}-\mathrm{left}}$ 29.        **End if**
30.        **If** $N_{\mathrm{neighbor}}$ is in the right half plane and $P_{\mathrm{neighbor}-\mathrm{right}}{<P}_{\mathrm{right}}$, **then** 31.          $P_{\mathrm{right}}\leftarrow P_{\mathrm{neighbor}-\mathrm{right}}$ 32.          $N_{\mathrm{next}-\mathrm{right}}\leftarrow N_{\mathrm{neighbor}-\mathrm{right}}$ 33.        **End if**
34.      **End for** 35.       **If** $P_{\mathrm{left}}\leq P_{\mathrm{right}}$, **then** 36.           $N_{\mathrm{next}}\leftarrow N_{\mathrm{next}-\mathrm{right}}$ 37.       **Else** 38.          $N_{\mathrm{next}}\leftarrow N_{\mathrm{next}-\mathrm{left}}$ 39.       **End if** 40.       Update p and forward p to $N_{\mathrm{next}}$ 41.     **End if** 42.   **End if** 43. **End while**

### 4.8. Algorithm Complexity

For the W-PAGPSR in the greedy forwarding strategy, the neighbor nodes under the restricted range were first determined, and the relay nodes were then determined by calculating the weight of each node. Assuming that the number of neighbor nodes in the reliable communication range is m, it was obvious that m≤n. By calculating and comparing the weights of m nodes, the neighbor node with the largest greedy weight was selected as the next hop node to forward the packet, and we then selected the neighbor node with the largest greedy weight as the next hop node to forward the packet. Therefore, for each node, the time complexity of the greedy forwarding strategy in W-PAGPSR was O(n). For the entire network, the time complexity of W-PAGPSR’s greedy forwarding strategy was ${O(n}^{2})$. W-PAGPSR needed to establish a cross-free network in the perimeter forwarding strategy. In a crossover-free network, the sending node selected the node with the largest perimeter weight as the next hop node. Therefore, for each node, the time complexity of the perimeter forwarding strategy in W-PAGPSR was ${O(n}^{2})$. For the entire network, the time complexity perimeter forwarding strategy in W-PAGPSR was ${O(n}^{3})$. Table 4 shows the time complexity comparison of W-PAGPSR, GPSR, MMGPSR, and PA-GPSR.

## 5. Simulation and Performance Analysis

Network Simulator 3 (NS3) is an open-source network simulator designed specifically for network simulation and research. It supports the use of common network protocols, such as TCP, UDP, IP, and Ethernet, to implement and evaluate various network solutions and algorithms. Through its rich functionality and visualization tools, NS3 helps researchers and developers better understand and evaluate the performance of network protocols, algorithms, and applications. With NS3, users can create and customize various network simulations, utilizing C++libraries, modules, and APIs for rapid development and experimentation. At the same time, the NS3 provides logging and output file setting functions, allowing users to analyze and evaluate simulation results to gain a deeper understanding of network behavior and performance.

SUMO is an open-source software package used to simulate urban traffic flow. It is a widely used tool in traffic simulation and research, which can be used to simulate and evaluate various traffic scenarios, traffic management strategies, and traffic flow patterns. Users can use SUMO’s built-in map editor or import existing map data to build a road network model. SUMO can simulate the traffic flow situation in actual traffic environments to generate realistic traffic flow patterns.

Use SUMO and NS3 to simulate GPSR, MM-GPSR, PA-GPSR, and W-PAGPSR in different node count and source-destination node count scenarios.

### 5.1. Simulation Parameter Setting

**Figure 8:**
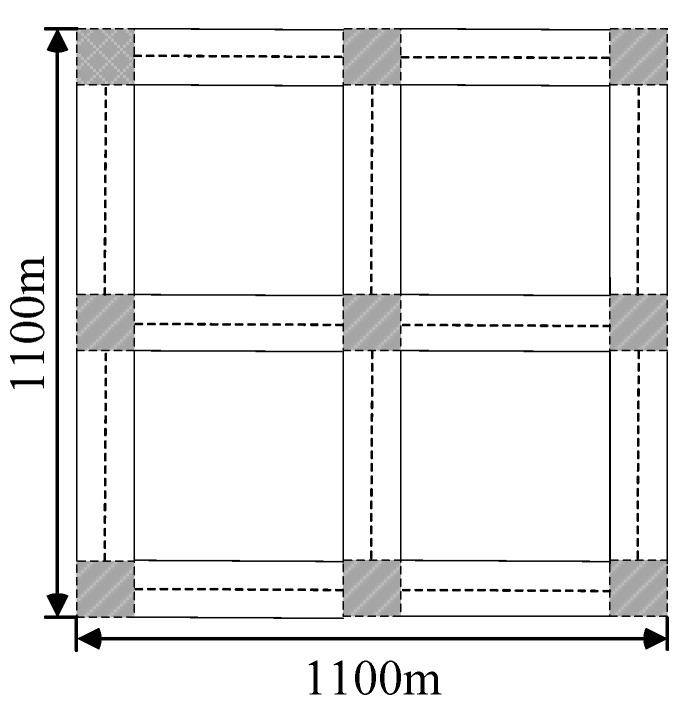
A simulation scene diagram, in which the gray patterned boxes represent the intersections, and the dotted lines represent the lane boundaries.

All simulations are based on NS-3.27. Using the Manhattan grid, as shown in Figure 8, the simulation area of 1100×1100 m^2^ consists of 9 intersections and 12 two-way streets. We imported this data into SUMO to generate realistic movement trajectories. The initial position of the node obeys a random distribution, the movement of the node on the street is limited by the Krauss model, and the maximum speed of the node is 15 m/s. We generated several motion tracking files that were input into our NS3 module. Trace files were used in NS3 to generate different VANET communication scenarios. All vehicles had the same physical configurations. We implemented this approach using IEEE 802.11p MAC with a channel data rate of 3 Mb/s. The MAC layer did not have any QoS. The MAC layer used the TwoRayGround propagation model to measure the wireless signal fading characteristics.

The number of vehicles varies between 30 and 110. For communication, it is assumed that each vehicle is equipped with wireless transceivers that comply with the IEEE 802.11p standard, which is applicable to multi hop propagation schemes. The maximum number of packets allowed to be buffered via the routing protocol is 64, and the maximum time allowed for buffering via the routing protocol is 30 s. The maximum communication radius of the node is set to 250 m. The location of the node can be obtained through accurate location services, and there is no wrong location information. At the beginning of each simulation, S and D are randomly generated and remain unchanged until the end of the simulation. At the beginning of each simulation process, nodes S and D are randomly determined. Node S uses UDP, and each connection generates a 512 byte packet every 0.2 s as a constant bit rate (CBR). Considering the impact of data traffic, we set the number of CBR connections to vary from 5 to 20 pairs to simulate VANET scenarios under different data traffic conditions [40]. The simulation of each scenario was run for 200 s, and all data results were averaged 30 times. According to CRITIC calculation results, during simulation, we set the greedy forwarding weight parameter to $w_{1}=0.4$, $w_{2}=0.3$, $w_{3}=0.2$, and $w_{4}=0.1$, and we set the perimeter forwarding weight parameter to $\beta_{1}=0.6$ and $\beta_{2}=0.4$. Other simulation parameters are shown in Table 5.

### 5.2. Result Analysis

To verify the performance of the proposed routing protocol in sparse urban network scenarios, we selected three key performance indicators to evaluate the performance of the protocol, which were packet loss rate, throughput, and average end-to-end delay [10]. The packet loss rate measures the effectiveness and stability of the protocol in terms of reliable data transmission. The lower packet loss rate means that data packets can be reliably transmitted from the source node to the target node. Throughput is one of the indicators used to measure routing efficiency. High-throughput routing protocols can support more data transmission and provide faster rates. The average end-to-end delay is an important indicator used to measure the delay of data transmission. In sparse urban network scenarios, reducing the delay is essential for real-time data exchange and application. By evaluating these three performance indicators, we can fully understand the performance of the proposed routing protocol in sparse urban network scenarios, so as to determine its reliability, efficiency, and applicability in meeting the communication needs in VANET.

(1) Packet loss rate: the ratio of the total number of lost data packets $P_{N_{s}}$ to the total number of data packets P_L_.

(19) $$Packet\;loss\;rate=\frac{P_{N_{s}}}{P_{L}}\times 100$$

(2) Throughput: the amount of information successfully transmitted per unit time via the communication channel. The greater the throughput, the shorter the time the algorithm requires to send the same number of data packets.

(20) $$Throughput=\frac{P_{N_{d}}}{T}$$

(3) Average end-to-end delay: the average of the delay D_n_ of all successfully received packets. The MAC protocol affects the transmission delay, and the use of the standard 802.11PMAC protocol is considered in the [41].

(21) $$End-to-end\;latency=\frac{\sum_{1}^{n}D_{n}}{n}$$

#### 5.2.1. Packet Loss Rate

**Figure 9:**
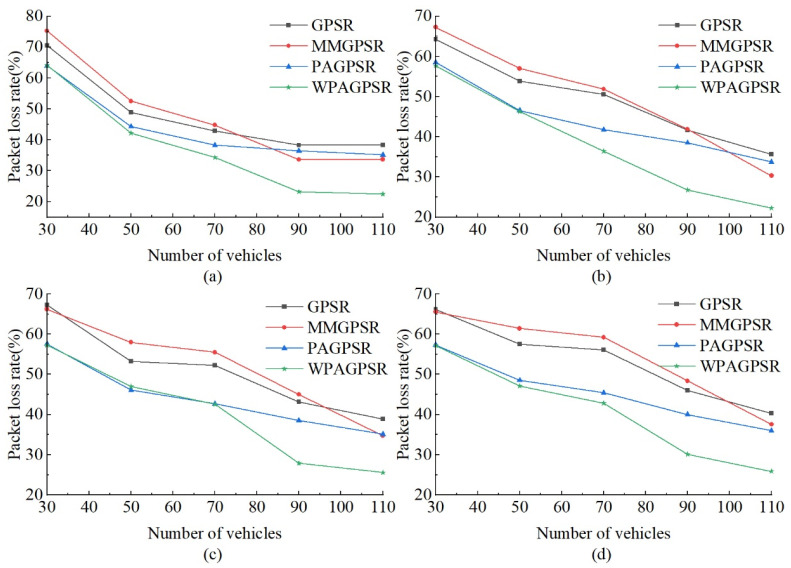
Packet loss rate under different number of nodes and CBR connections; (**a**) 5 CBR connections; (**b**) 10 CBR connections; (**c**) 15 CBR connections; (**d**) 20 CBR connections.

Figure 9 shows the packet loss rate of each routing protocol when changing the number of nodes and CBR connections. As the number of nodes increases, the number of reliable links and network connectivity increase, thereby increasing the probability of data packets successfully reaching the destination node. On the whole, when the number of nodes increases, the packet loss rate of all protocols will decrease. Considering the distance, direction of movement, reliable node density, and cumulative communication duration required to select the next hop node, the probability of W-PAGPSR successfully sending data packets is higher. In a network environment with several nodes from 30 to 110, data packets are more likely to be discarded in the process of finding the correct path. As the number of nodes increases, the number of neighbor nodes available for decision-making increases; thus, the packet loss rate of W-PAGPSR is lower than those of other protocols. In addition, each data packet has a lifetime to stay in the network, which reduces the probability of routing loops occurring in sparse networks, thereby reducing the packet loss rate.

As shown in Figure 9a, with five CBR connections, the packet loss rate of all nodes of W-PAGPSR is lower than those of GPSR, MM-GPSR, and PA-GPSR, respectively. As shown in Figure 9a, with five CBR connections, the packet loss rate of all nodes of W-PAGPSR is lower than those of MM-GPSR, PA-GPSR, and GPSR, respectively. As shown in Figure 9a, the average packet loss rate of W-PAGPSR is 24.68%, 24.41%, and 17.48% lower than those of GPSR, MM-GPSR, and PA-GPSR, respectively. Neither GPSR nor MM-GPSR considers the link quality in the process of selecting the next hop; thus, the packet loss rate is higher. MM-GPSR selects the next hop by accumulating the communication time. This choice is not the nearest hop to the destination, which leads to an increase in the number of hops and increases the probability of packet loss by the node.

From Figure 9b, it can be seen that the packet loss rate of the four protocols at 70 nodes has increased. As shown in Figure 9b, the average packet loss rate of W-PAGPSR is 25.09%, 25.08%, and 15.87% lower than those of GPSR, MM-GPSR, and PA-GPSR, respectively. This result occurs because when the number of CBR connection pairs increases, the network load increases and a large number of nodes access similar links, increasing the chance of collision and interference between data packets, thus increasing the packet loss rate. When the number of nodes reaches 110, W-PAGPSR performance tends to stabilize because it balances the load between nodes in the network, which helps to make more efficient use of available bandwidth and, ultimately, deliver more data packets. As shown in Figure 9c, the average packet loss rate of W-PAGPSR is 22.98%, 244.08%, and 10.77% lower than those of GPSR, MM-GPSR, and PA-GPSR, respectively. As shown in Figure 9d, the average packet loss rate of W-PAGPSR is 25.14%, 26.53%, and 12.38% lower than those of GPSR, MM-GPSR, and PA-GPSR, respectively. Overall, the packet loss rate of W-PAGPSR in sparse scenarios is smaller than those of GPSR, MM-GPSR, and PA-GPSR. This result occurs because W-PAGPSR chooses the next hop with a more stable connection, a smaller distance to the destination, and better connectivity.

As shown in Figure 9, judging from the overall trend, from 30 to 70 nodes, the packet loss rate advantage of W-PAGPSR is not obvious. This result occurs because the number of nodes is insufficient, and the improvement strategy of W-PAGPSR cannot take advantage of the advantages of multi-parameter selection of relay nodes. From 70 to 90 nodes, the packet loss rate of W-PAGPSR has obvious advantages. This result occurs because a sufficient number of nodes can use improved greedy forwarding and perimeter forwarding methods for routing decisions, thereby reducing the packet loss rate. From 90 to 110 nodes, the decline in packet loss rate slows down. This result occurs because the probability of data congestion caused by higher node density increases. At the same time, W-PAGPSR has packet survival time and MAC queue size, which leads to the loss of some data packets and increases the packet loss rate.

#### 5.2.2. Throughput

**Figure 10:**
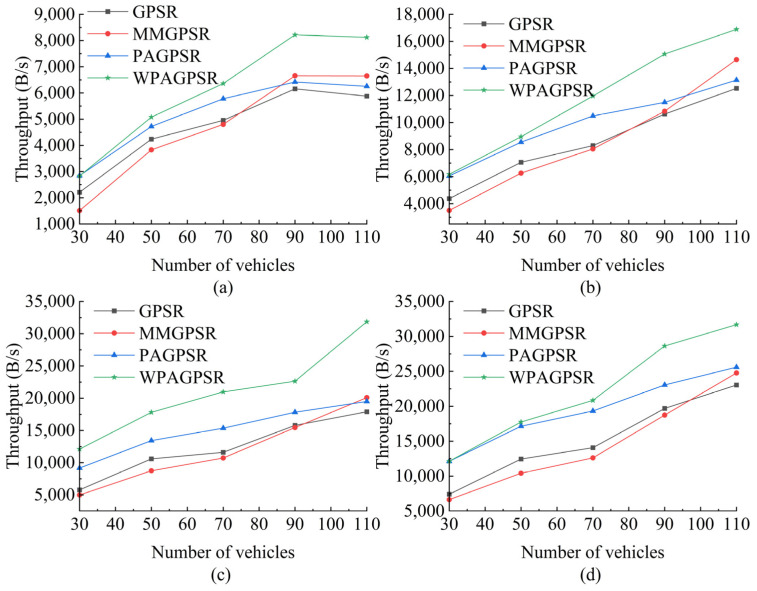
The throughput under different number of nodes and CBR connections: (**a**) 5 CBR connections; (**b**) 10 CBR connections; (**c**) 15 CBR connections; (**d**) 20 CBR connections.

**Figure 11:**
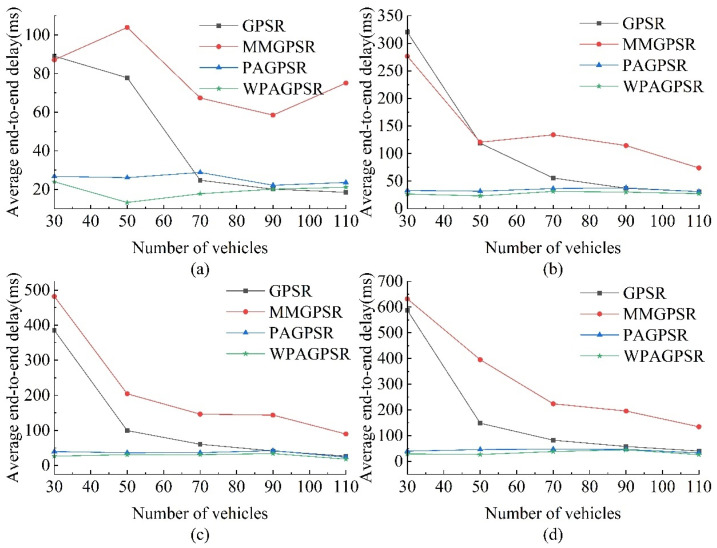
Average end-to-end delay rates under different numbers of nodes and CBR connections: (**a**) 5 CBR connections; (**b**) 10 CBR connections; (**c**) 15 CBR connections; (**d**) 20 CBR connections.

Figure 10 shows the throughput of each routing protocol when changing the number of nodes and CBR connections. When the number of nodes increases, the throughput performance is improved. This result occurs because the connectivity between nodes increases with the increase in nodes, reducing the rate of redundant packet transmission and packet loss. The main reason for limiting the performance of routing protocols in sparse networks is insufficient network connections caused by the small number of nodes. The location-based greedy forwarding strategy is very suitable for well-connected networks because the scheme aims to obtain the least number of hops. In Figure 9 and Figure 11, it can be seen that W-PAGPSR has a lower packet loss rate and lower end-to-end delay. Compared to GPSR, MM-GPSR, and PA-GPSR under all CBR connections, the W-PAGPSR proposed in this paper has higher throughput in sparse environments. The proposed routing protocol uses a variety of indicators as routing measures during packet transmission; this, the node can estimate the link stability and select the most stable relay node during packet transmission.

As shown in Figure 10a, the average throughput of W-PAGPSR is 29.61%, 36.70%, and 15.01% higher than those of GPSR, MM-GPSR, and PA-GPSR, respectively. Figure 10b shows that the average throughput of W-PAGPSR is 37.72%, 44.45%, and 16.05% higher than those of GPSR, MM-GPSR, and PA-GPSR, respectively. As shown in Figure 10c, the average throughput of W-PAGPSR is 75.89%, 89.33%, and 38.36% higher than those of GPSR, MM-GPSR, and PA-GPSR, respectively. Figure 10d shows that the average throughput of W-PAGPSR is 47.47%, 59.89%, and 11.89% higher than those of GPSR, MM-GPSR, and PA-GPSR, respectively. Since W-PAGPSR considers link quality and uses a weight model when selecting the next hop forwarding node, it shows better network resource utilization than other protocols.

As shown in Figure 10, from the overall trend point of view, from 30 to 70 nodes, the throughput advantage of W-PAGPSR is not obvious, which is because the insufficient number of nodes leads to a small number of packets successfully reaching the destination node. From 70 to 90 nodes, the throughput advantage of W-PAGPSR is obvious. This result occurs because the use of improved greedy forwarding and perimeter forwarding strategies to select relay nodes reduces the number of data retransmissions, which saves network bandwidth and can be used to transmit more data packets, thereby improving network throughput. From 90 to 110 nodes, the increase in throughput slows down. This result occurs because the probability of data congestion caused by higher node density increases, which increases the packet loss rate.

#### 5.2.3. Average End-to-End Delay

Figure 11 shows the average end-to-end delay of each routing protocol when changing the number of nodes and CBR connections. The increase in the number of nodes participating in packet forwarding reduces the probability of link interruption, thereby reducing the average end-to-end delay of each routing protocol. In the process of transmitting data, the end-to-end delay is mainly determined based on the number of hops. When the nodes are sparse, data packets frequently enter the perimeter forwarding mode, resulting in a higher number of hops in the route; thus, the end-to-end delay performance at 70 nodes is significantly better than at 30 nodes.

For GPSR and MM-GPSR, due to the lack of full consideration of the network environment, data packets are likely to enter sparse sections with insufficient connections between nodes, and routing voids are more likely to be encountered. GPSR adopts a perimeter forwarding strategy more frequently; thus, it has a higher delay than other protocols. MM-GPSR uses the minimum packet delivery angle under the left-hand and right-hand rule for perimeter forwarding. PA-GPSR copies data packets and applies left-handed and right-handed rules for perimeter forwarding, which increases the possibility of finding forwarding nodes in sparse networks; thus, it has a lower delay at low densities. Based on PA-GPSR, W-PAGPSR uses the packet delivery angle and reliable node density as weight reference indicators for perimeter forwarding.

As shown in Figure 11a, the average end-to-end delay of W-PAGPSR is 34.04%, 74.18%, and 23.51% lower than those of GPSR, MM-GPSR, and PA-GPSR, respectively. Figure 11b shows that the average end-to-end delay of W-PAGPSR is 49.37%, 76.94%, and 18.57% lower than those of GPSR, MM-GPSR, and PA-GPSR, respectively. As shown in Figure 11c, the average end-to-end delay of W-PAGPSR is 52.35%, 82.89%, and 21.25% lower than those of GPSR, MM-GPSR, and PA-GPSR, respectively. Figure 11d shows the average end-to-end delay of W-PAGPSR is 48.33%, 79.96%, and 21.45% lower than those of GPSR, MM-GPSR, and PA-GPSR, respectively. By selecting the next hop with high reliability, W-PAGPSR has a lower end-to-end delay than GPSR, MM-GPSR, and PA-GPSR.

As shown in Figure 11, from the overall trend point of view, because PA-GPSR’s packet control and forwarding strategy has greatly improved its average end-to-end delay compared to GPSR and MM-GPSR, W-PAGPSR has been improved on the basis of it, further reducing the average end-to-end delay while considering more parameters. From 70 to 110 nodes, the average short end-to-end delay of GPSR is similar to that of W-PAGPSR, but according to Figure 9, its packet loss rate is higher, and the index counts the average end-to-end delay of successfully transmitted data packets.

## 6. Conclusions

VANET is of great significance in practice, as it can improve traffic safety, optimize traffic efficiency, and support intelligent transportation systems. Efficient and reliable routing protocols play a crucial role in VANET, providing stable and reliable data transmission by selecting the best relay vehicle and adapting to dynamic topology changes. To improve the local optimality and routing loops of PA-GPSR in urban sparse networks, W-PAGPSR is proposed. Given the geographic location routing protocols in the VANET network, this paper applies weight-based improvement strategies to routing establishment and routing maintenance to improve the routing stability of data packet transmission. The main contributions of this paper can be summarized as follows:

(1) Firstly, in the routing establishment stage, the distance, reliable node density, cumulative communication duration, and node movement direction are used to establish a greedy forwarding weight strategy; secondly, in the routing maintenance stage, the packet delivery angle and reliable node density are used to establish a perimeter forwarding weight strategy, and the weight parameters are then calculated using the CRITIC method; and, finally, a highly reliable link is established as a communication route.
(2) Based on the NS-3 and SUMO simulation platforms, the two strategies are simulated and compared to other geographic location routing protocols in urban scenarios with different traffic densities and CBR connections. Compared to GPSR, MM-GPSR, and PA-GPSR in 30–110 nodes and 5–20 CBR connections, the packet loss rate of the protocol is reduced by an average of 24.47%, 25.02%, respectively; and 14.12%, the average end-to-end delay is reduced by an average of 48.34%, 79.96%, and 21.45%, respectively; and the network throughput is increased by an average of 47.68%, 58.39%, and 20.33% respectively.
(3) According to the time complexity analysis, compared to GPSR, MM-GPSR, and PA-GPSR, W-PAGPSR does not add to the time complexity.

The simulation results show that W-PAGPSR has a lower average end-to-end delay, a higher packet transmission rate, and overall network throughput. The results indicate that W-PAGPSR has the potential to improve the routing performance of urban VANET under different network densities.

The current research is based on the simulation of the urban Manhattan grid, though there are certain differences from the actual urban network. Future research can adjust and optimize the parameters of the proposed algorithm, consider applying the algorithm to dense urban and highway scenarios to more realistically simulate the urban traffic environment, and explore the application of the algorithm in other networks (such as underwater ad hoc and UAV ad hoc networks). The current research assumes that node position information is perfect, but, in practice, GPS positioning has errors. Future research can consider introducing GPS error models to more accurately simulate node position information, as well as investigate the impact of GPS errors on algorithm performance and how to respond to such error situations.

## Figures and Tables

**Table 1:** A summary of the reviewed location-based routing protocols.

Protocol	Scenario	Selection Parameters	Communication	Forwarding Mechanism	Recovery Mechanism	Performances Metrics	Sender/ Receiver Oriented
GPSR [14]	Highway	Distance	V2V	Greedy forwarding	Perimeter forwarding	Packet loss rate and overhead	Sender-oriented
GPSRJ+ [15]	Highway	Distance, direction, and speed	V2V	Improved greedy forwarding	Improved perimeter forwarding	Packet delivery rate, delay, throughput, and overhead	Sender-oriented
MM-GPSR [16]	Urban	Cumulative communication duration	V2V	Improved greedy forwarding	Improved perimeter forwarding	Packet loss rate, delay, and throughput	Sender-oriented
PA-GPSR [17]	Urban	Distance	V2V	Improved greedy forwarding	Improved perimeter forwarding	Packet loss rate, delay, and network yield	Sender-oriented
PGRP [18]	Urban and highway	Distance, direction, and angle	V2V	Predictive greedy forwarding	Predictive perimeter forwarding	Packet delivery rate, delay, and hop count	Sender-oriented
K-PGRP [19]	Urban and highway	Distance, direction, and angle	V2V	Predictive greedy forwarding	Predictive perimeter forwarding	Packet delivery rate and throughput	Sender-oriented
MPBRP [20]	Urban	Distance, direction, and angle	V2V	Predictive greedy forwarding	Predictive perimeter forwarding	Packet delivery rate, delay, and hop count	Sender-oriented
W-GPCR [21]	Urban	Distance, direction, and density	V2V	Improved greedy forwarding	Improved perimeter forwarding	Packet delivery rate, delay, and hop count	Sender-oriented
HRNS [22]	Highway	Distance, traffic load, speed, and density	V2V	Hybrid relay node selection scheme	_	Reachability, delay, and saved rebroadcast	Sender-oriented
Geo-LU [23]	Urban	Residual bandwidth and link quality	V2V	Two-hop information-based greedy forwarding	_	Packet delivery rate, throughput, and overhead	Sender-oriented
TGRV [24]	Urban	Distance, speed, direct trust, recommendation trust, and direction	V2V	Trust-based greedy forwarding	Trust-based perimeter forwarding	Packet delivery rate, delay, and hop count	Sender-oriented
SFTD [25]	Urban	Link quality	V2V	Smart data dissemination strategy	_	Packet delivery rate, delay, and throughput	Receiver -oriented
ReUse [26]	Highway	Mobility, link quality, buffer size, and the number of neighbors	V2V, V2I, and I2I	Relay selection using harmony search and fuzzy analytic hierarchy process	_	Packet delivery rate, packet sent rate, delay, reachability, collision rate, redundancy rate, and throughput	Receiver -oriented
OPBRP [27]	Urban	Distance, direction, position, and speed	V2V and V2I	Predictive greedy forwarding	Predictive perimeter forwarding	Packet delivery rate, delay, power consumption, and hop count	Sender-oriented
Geo-CAP [28]	Urban	Bandwidth availability and link quality	V2V	Improved greedy forwarding	Carry-and-forward strategy	Packet delivery rate, delay, and throughput	Sender-oriented
MCBS [29]	Urban and highway	Distance, density, angle, link stability, and velocity	V2V	Contention-based forwarding	_	Packet delivery rate, delay, hop count, and throughput	Receiver-oriented
REMR [30]	Highway	Position, distance, speed, and moving angle	V2V	Improved greedy forwarding	Carry-and-forward strategy	Packet delivery rate, delay, and hop count	Sender-oriented
MISP [31]	Urban	Distance, density, road connectivity, and link stability	V2V	Multiple intersection selection routing algorithm	_	Packet delivery rate and delay	Sender-oriented
TLBGR [32]	Urban	Density, road connectivity, and link stability	V2V and V2I	Next link segment selection and next hop selection based on trunk line	_	Packet delivery rate, delay, and overhead	Sender-oriented
ISR [33]	Urban	Distance, density, direction, and link stability	V2V	Information dissemination-centric routing	_	Packet delivery rate, delay, and throughput	Sender-oriented

**Table 2:** A HELLO packet header format table.

Name	Size
The package type	2 bytes
The x co-ordinate of the sender	8 bytes
The y co-ordinate of the sender	8 bytes
The number of neighbors	4 bytes

**Table 3:** A format table of packet header.

Name	Size
The package type	2 bytes
The x co-ordinate of the destination	8 bytes
The y co-ordinate of the destination	8 bytes
The exact time when the location was last updated	4 bytes
The x co-ordinate of entering the perimeter mode	8 bytes
The y co-ordinate of entering the perimeter mode	8 bytes
The perimeter mode flag	1 byte
The x co-ordinate of the previous hop	8 bytes
The y co-ordinate of the previous hop	8 bytes

**Table 4:** A time complexity comparison table.

	Greedy Forwarding	Perimeter Forwarding
GPSR	${O(n}^{2})$	${O(n}^{3})$
MMGPSR	${O(n}^{2})$	${O(n}^{3})$
PA-GPSR	${O(n}^{2})$	${O(n}^{3})$
W-PAGPSR	${O(n}^{2})$	${O(n}^{3})$

**Table 5:** A simulation parameter setting table.

Parameter	Value
Data packet size/B	512
Simulation time/s	200
Simulation area/m^2^	1100 × 1100
Number of nodes	30, 50, 70, 90, 110
HELLO interval/s	1
Number of CBR connections	5, 10, 15, 20
Transport protocol	UDP
Maximum speed/(m·s^−1^)	15
Channel data rate/(Mbit·s^−1^)	3
MAC protocol	IEEE 802.11p
Packet interval/s	0.2

## Data Availability

Not applicable.
